# Efficacy of a mitochondrion-targeting agent for reducing the level of urinary protein in rats with puromycin aminonucleoside-induced minimal-change nephrotic syndrome

**DOI:** 10.1371/journal.pone.0227414

**Published:** 2020-01-06

**Authors:** Yuko Fujii, Hideki Matsumura, Satoshi Yamazaki, Akihiko Shirasu, Hyogo Nakakura, Tohru Ogihara, Akira Ashida

**Affiliations:** 1 Department of Pediatrics, Osaka Medical College, Takatsuki, Osaka, Japan; 2 Department of Pediatrics, Hirakata City Hospital, Hirakata, Osaka, Japan; 3 Department of Hemodialysis and Apheresis, Arisawa General Hospital, Hirakata, Osaka, Japan; National Institutes of Health, UNITED STATES

## Abstract

**Background:**

Oxidative stress is a major factor responsible for minimal-change nephrotic syndrome (MCNS), which occurs most commonly in children. However, the influence of oxidative stress localized to mitochondria remains unclear. We examined the effect of a mitochondrion-targeting antioxidant, MitoTEMPO, in rats with puromycin aminonucleoside (PAN)-induced MCNS to clarify the degree to which mitochondrial oxidative stress affects MCNS.

**Materials and methods:**

Thirty Wistar rats were divided into three groups: normal saline group (n = 7), PAN group (n = 12), and PAN + MitoTEMPO group (n = 11). Rats in the PAN and PAN + MitoTEMPO groups received PAN on day 1, and those in the PAN + MitoTEMPO group received MitoTEMPO on days 0 to 9. Whole-day urine samples were collected on days 3 and 9, and samples of glomeruli and blood were taken for measurement of lipid peroxidation products. We also estimated the mitochondrial damage score in podocytes in all 3 groups using electron microscopy.

**Results:**

Urinary protein excretion on day 9 and the levels of lipid peroxidation products in urine, glomeruli, and blood were significantly lower in the PAN　+　MitoTEMPO group than in the PAN group (*p* = 0.0019, *p* = 0.011, *p* = 0.039, *p* = 0.030). The mitochondrial damage score in podocytes was significantly lower in the PAN　+　MitoTEMPO group than in the PAN group (*p* <0.0001).

**Conclusions:**

This mitochondrion-targeting agent was shown to reduce oxidative stress and mitochondrial damage in a MCNS model. A radical scavenger targeting mitochondria could be a promising drug for treatment of MCNS.

## Introduction

It has recently become evident that mitochondrial damage is associated with a wide range of conditions affecting the brain [1], cardiovascular system [2], lungs [3], digestive tract [4] and liver [5], as well as in metabolic diseases and neoplasia [6]. Mitochondrial damage is also implicated in various conditions affecting the kidney, such as acute kidney injury due to sepsis [7], chemical agents [8] or other substances [9, 10], congenital nephrotic syndrome [11], focal segmental glomerulosclerosis [12–15], diabetic nephropathy [16], acute or chronic interstitial nephritis [17], chronic kidney disease [18], and transplanted kidney grafts [19, 20].

(2-(2,2,6,6-Tetramethylpiperidin-1-oxyl-4-ylamino)-2-oxoethyl) triphenylphosphonium chloride (MitoTEMPO) is a mitochondrion-targeting radical scavenger [2, 7, 20–23]. The positive charge carried by the triphenylphosphonium component enables MitoTEMPO to pass through the lipid bilayer into the mitochondrial matrix, where it accumulates to a concentration 1000-fold higher than in other areas [23]. Mitochondria consume as much as approximately 85% of the total amount of oxygen required by the cell to produce adenosine triphosphate, at the same time producing large amounts of reactive oxygen species (ROS) and lipid oxidation products [24]. As the TEMPO component of MitoTEMPO scavenges ROS such as superoxide dismutase, it can improve the status of many mitochondrion-associated diseases [23].

Rats given a single injection of puromycin aminonucleoside (PAN) constitute a well-established model of minimal-change disease [25]. The pathology of PAN-induced minimal-change nephrotic syndrome (MCNS) has been explained in terms of podocyte damage and apoptosis due to a ROS surge [25, 26]. We have previously reported that alpha-tocopherol and edaravone, radical scavengers that inhibit lipid peroxidation, reduce ROS in rats with PAN-induced MCNS [27, 28]. Recently, it has become evident that α-tocopherol and edaravone also exert a favorable effect on mitochondria [29, 30]. These observations, as well as the fact that mitochondria produce large amounts of ROS, suggest that PAN-induced MCNS may be intimately associated with mitochondrial damage.

Although a few reports have suggested that mitochondrial damage may underlie MCNS, to our knowledge those studies all involved an *in vitro* approach [31, 32]. In the present *in vivo* study, therefore, we investigated whether mitochondrial damage is one of the major causes of PAN-induced MCNS.

## Materials and methods

### Ethics statement

All of the present experiments were conducted in accordance with the protocol for treatment of experimental animals at Osaka Medical College. The experimental protocol, including ethical aspects, was approved by the Osaka Medical College Animal Care and Use Committee (approval numbers: 28054, 29057). All necropsy procedures were performed soon after euthanasia with an overdose of intraperitoneal sodium pentobarbital and all efforts were made to minimize suffering.

### Reagents

PAN and MitoTEMPO were purchased from Sigma-Aldrich, St. Louis, MO, USA. PAN was diluted in 0.9% normal saline (NS) to a concentration of 10 mg/mL at the time of use. MitoTEMPO was diluted in 0.9% NS to a concentration of 1.0 mg/mL on day 0, stored at 4°C, and on day 2 it was diluted to 0.2 mg/mL in 0.9% NS.

### Experimental animals

Thirty male Wistar rats, 6 weeks of age, weighing 120–150 g (Japan SLC, Shizuoka, Japan) were housed in metabolic cages and given free access to commercial feed (Oriental Yeast, Tokyo, Japan) and water. They were divided into three groups: NS group (n = 7), PAN group (n = 12), and PAN + MitoTEMPO group (n = 11). PAN (5 mg/100 g body weight) was injected subcutaneously on day 1 [27]. MitoTEMPO was injected intraperitoneally at a dose of 0.5 mg/100 g body weight on days 0 to 1, and then at 0.07 mg/100 g body weight on days 2 through 9 [2, 7, 22]. 0.9% NS was used as a placebo. Whole-day urine samples were collected from these rats on days 3 and 9, and stored at -80°C. The rats were sacrificed on day 10, and samples of glomeruli and blood plasma were taken and stored at -80°C. These samples were used for measurement of protein, creatinine, and 2-thiobarbituric acid-reactive substances (TBARS), as reported previously [27]. We calculated creatinine clearance (mL/min/kg) from the data according to the formula: (UV x Ucr) / (Pcr x 1000), where UV = urine volume (μL/min/kg), Ucr = urinary creatinine (mg/dL), and Pcr = plasma creatinine (mg/dL). Randomly obtained plasma samples (n = 4, 4, 4, respectively) were also subjected to measurement of 4-hydroxynonenal (4HNE) concentration using negative-ion chemical ionization gas chromatography-mass spectrometry as oxime-tert-butyldimethylsilyl derivatives with an internal standard of 2,2,6,6-d4-cyclohexanone using a Shimadzu QP-5050A system (Shimadzu, Kyoto, Japan), as reported previously [33]. As another marker of oxidative stress, the plasma dityrosine concentration (n = 7, 8, 8) was also measured from randomly chosen samples, as described previously [33]. The data were expressed as ratios relative to the parent molecule, para-tyrosine (nmol/μmol x 10^2^).

### Light microscopy and immunohistochemical analysis

Upon sacrifice on day 10, the left kidney was removed from each rat. Paraffin-embedded sections of formalin-fixed kidney tissue were prepared after deparaffinization in xylene. Some were subjected to periodic acid-Schiff staining using a standard procedure, and others were used for immunohistochemical analysis of 4HNE or cleaved caspase-3. The latter were rehydrated by serial immersion in 100% ethanol, 90% ethanol, 70% ethanol, and water, then antigen retrieval was conducted by heating in a microwave oven followed by washing with phosphate-buffered saline (PBS). Endogenous peroxidase activity was reduced by immersion in 3% hydrogen peroxide. After rinsing, sections were covered in 3% goat serum for 30 minutes and incubated with 25 μg/mL murine-derived anti-4-HNE monoclonal antibody (NIKKEN SEIL, Tokyo, Japan) diluted in PBS (1:50) overnight. After incubation, the sections were rinsed with PBS and incubated with biotin-labeled secondary antibody diluted 1: 300 in PBS for 2 hours. After incubation with the secondary antibody, the sections were rinsed with PBS, incubated in ABC complex (Vector Laboratories, Burlingame, CA, USA) for 30 minutes at room temperature, rinsed in PBS, and developed with diaminobenzidine and hydrogen peroxide. Slides were lightly counterstained with hematoxylin, then dehydrated by sequential immersion in 70% ethanol, 90% ethanol, 100% ethanol, and xylene before applying coverslips. For assessment of glomeruli, each section was visualized using a Nikon Eclipse 80i microscope with a ×40 objective and captured as a TIFF image using a DS-Ri1 digital camera (Nikon, Tokyo, Japan). We observed all of the glomeruli visible on each slide to evaluate the location of peroxidation. For cleaved caspase-3 staining, we employed the same protocol using a 2000-fold diluted anti-cleaved caspase-3 antibody (Cell Signaling Technology, Denvers, MA, USA) as the primary antibody. All glomeruli from each group, as far as we could observe using the light microscope, were photographed to count the number of positive cells in each glomerulus.

### Electron microscopy

Part of the left kidney from two different rats in the PAN group and PAN + MitoTEMPO group, and a single rat in the NS group, was randomly chosen, prefixed in 2% glutaraldehyde in 0.1M PBS (pH 7.4) for 3 hours, then post-fixed in 1% osmium tetroxide in 0.1M PBS at 4°C for 2 hours. The fixed samples were washed five times in 0.1M PBS and dehydrated through a graded ethanol series for 15 minutes each. They were then embedded in epoxy resin and processed using a regular procedure for electron microscopy. Ultrathin sections were cut with an ultramicrotome (ULTRACUT-N, Reichert-Nissei, Tokyo, Japan) and mounted on a 200-mesh copper grid supported by a carbon-coated collodion film. They were then double-stained with uranyl acetate and lead citrate. A non-expert in nephrology unrelated to the present study observed the specimens with a transmission electron microscope (H-7650 types, Hitachi, Tokyo, Japan) and took photographs of randomly chosen podocytes (n = 5, 18, 10, respectively) and their foot processes.

We then used the images to score the degree of mitochondrial damage for all of the mitochondria visible in each podocyte (n = 4–41 per podocyte) and calculated the average score for each podocyte. The method used in the present study for scoring of mitochondrial damage was reported previously by Sesso *et al*. [34] and Sweetwyne *et al*. [35], who observed mitochondria in preapoptotic and apoptotic cells using electron microscopy and categorized the mitochondria into 5 groups. Score 1: No obvious damage, clear inner and outer membranes with a dense matrix and regularly aligned cristae. Score 2: Crista membranes disrupted with occasional vacuolation, the matrix mostly remaining dense. Apparent separation of the inner and outer mitochondrial membranes. Score 3: Mitochondrial matrix largely diminished with few remaining cristae. Score 4: Cristae mostly absent. Matrix either absent or very electron dense, and evident rupture of some outer membranes. Score 5: Triple and quadruple membrane rings indicative of autophagy or mitophagy evident.

### Statistical analysis

The data were expressed as the median and interquartile range (IQR). We used the Kruskal-Wallis test and the Wilcoxon test to compare differences among the groups, and *p* <0.05 was considered the level of significance. All statistical analyses were performed using JMP Pro 13 software (SAS Institute, Cary, NC, USA).

## Results

### Daily urinary protein

**Fig 1:**
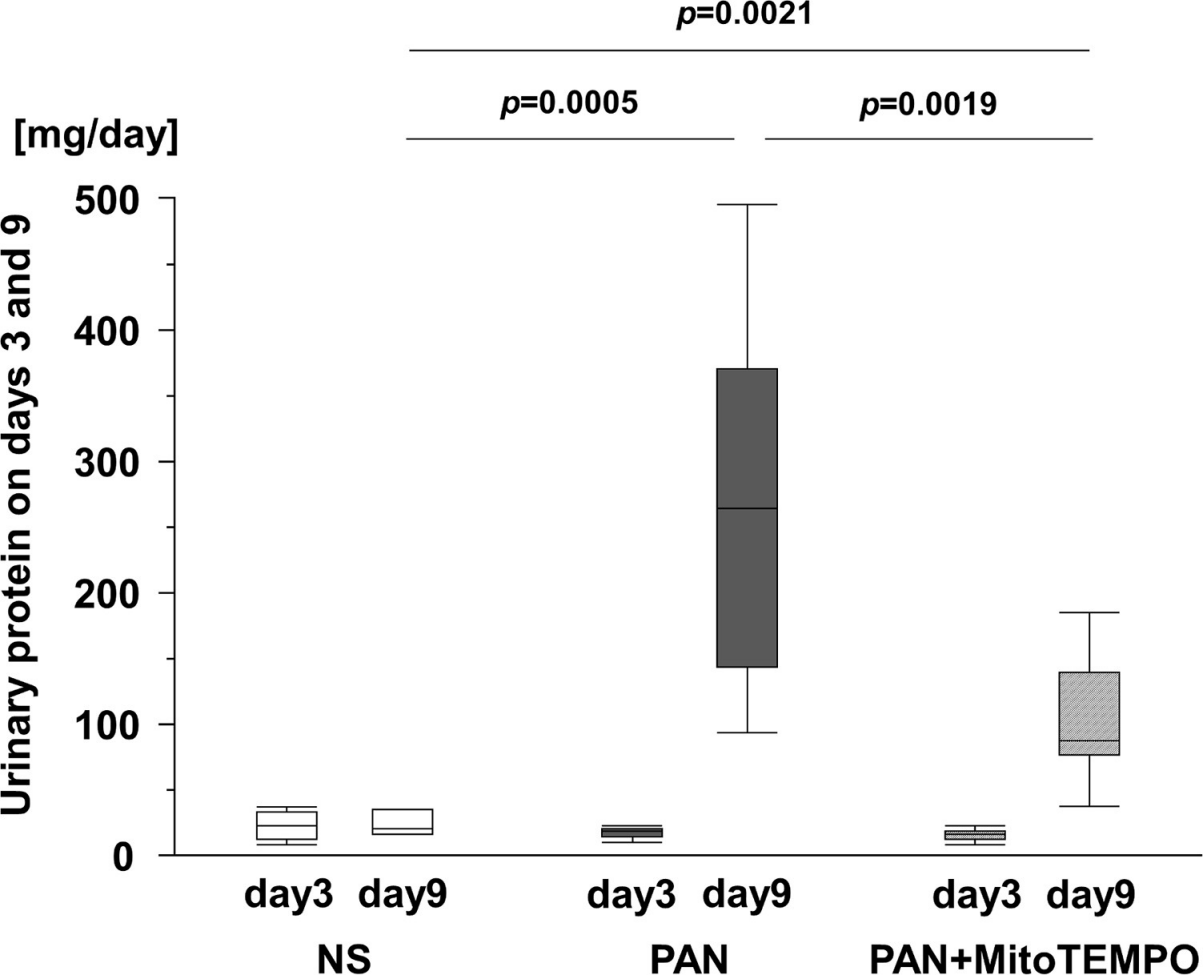
Daily urinary protein excretion on days 3 and 9. Thirty Wistar rats were divided into three groups: normal saline group (n = 7), PAN group (n = 12), and PAN + MitoTEMPO group (n = 11). Rats in the PAN and PAN + MitoTEMPO groups received 5 mg/100 g body weight subcutaneous PAN on day 1, inducing minimal-change nephrotic syndrome. Those in the PAN + MitoTEMPO group received intraperitoneal MitoTEMPO, a mitochondrion-targeting agent, at a dose of 0.5 mg/100 g body weight on days 0 to 1, and then at 0.07 mg/100 g body weight on days 2 through 9. Whole-day urine samples were collected from these rats on days 3 and 9. MitoTEMPO significantly reduced daily urinary protein excretion on day 9. Data are expressed as the median and inter-quartile range. PAN = puromycin aminonucleoside; NS = normal saline; n.s. = not significant.

In the present study, the daily level of urinary protein increased from days 3 to 9 in both the PAN group and the PAN + MitoTEMPO group (Fig 1). The level of daily urinary protein on day 3 showed no significant differences among the groups, but on day 9 it was 20.5 (IQR 16.2–35.7), 265.3 (IQR 144.0–371.7), and 87.5 (IQR 76.1–138.7) mg/day in the NS, PAN, and PAN + MitoTEMPO groups, respectively, being significantly lower (*p* = 0.0019) in the PAN + MitoTEMPO group than in the PAN group (Fig 1).

### Kidney function

The levels of creatinine clearance at sacrifice were 113.9 (IQR 75.4–168.7), 100.7 (IQR 73.8–125.7), and 76.5 (IQR 65.8–118.3) mL/min/kg in the NS, PAN, and PAN + MitoTEMPO groups, respectively, showing no significant differences among the groups.

### Lipid peroxidation

**Fig 2:**
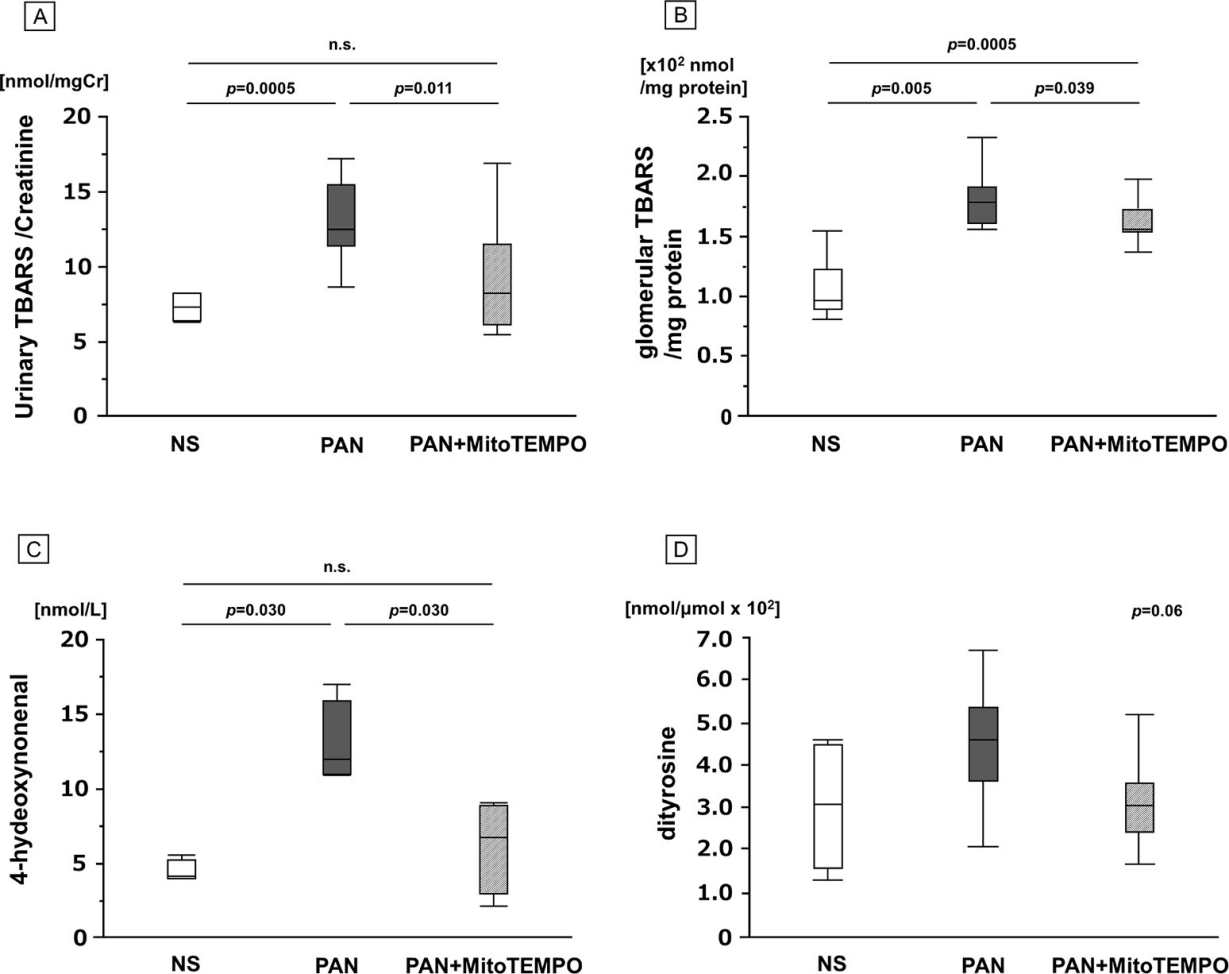
Oxidative stress markers. Reactive oxygen species at sacrifice in the NS, PAN, PAN + MitoTEMPO groups. **(A)** The levels of urinary TBARS/creatinine (nmol/mgCr) were measured by the method of Yagi. **(B)** The levels of glomerular TBARS (x10^2^ nmol/mg protein) were measured by the method of Beug and Aust. **(C)** The levels of plasma 4HNE (nmol/L) and **(D)** plasma dityrosine as ratios relative to the parent molecule, para-tyrosine (nmol/μmol x 10^2^), in randomly chosen samples (NS, PAN, PAN + MitoTEMPO; n = 4, 4, 4, and n = 7, 8, 8, respectively) were measured using negative-ion chemical ionization gas chromatography-mass spectrometry. Reactive oxygen species in rats with PAN-induced minimal-change nephrotic syndrome were decreased by treatment with MitoTEMPO. Data are expressed as the median and inter-quartile range. PAN = puromycin aminonucleoside; TBARS = 2-thiobarbituric acid-reactive substances; 4HNE = 4-hydroxynonenal; NS = normal saline; n.s. = not significant.

The levels of urinary TBARS on day 9 relative to creatinine were significantly lower (*p* = 0.011) in the PAN + MitoTEMPO group (8.3, IQR 6.1–11.5 nmol/mgCr) than in the PAN group (12.5, IQR 11.4–15.5 nmol/mgCr) (Fig 2A). The levels of glomerular TBARS relative to protein were significantly lower (*p* = 0.039) in the PAN + MitoTEMPO group (1.5, IQR 1.2–1.6 x10^2^ nmol/mg protein) than in the PAN group (1.8, IQR 1.6–1.7 x10^2^ nmol/mg protein) (Fig 2B). The levels of plasma 4HNE at sacrifice in randomly chosen samples were significantly lower (*p* = 0.030) in the PAN + MitoTEMPO group (6.8, IQR 3.0–8.9 nmol/L) than in the PAN group (12.0, IQR 11.0–16.0 nmol/L) (Fig 2C). The levels of plasma dityrosine at sacrifice in randomly chosen samples tended to be lower (*p* = 0.06) in the PAN + MitoTEMPO group (3.1, IQR 2.5–3.58 nmol/μmol x 10^2^) than in the PAN group (4.6, IQR 3.7–5.4 nmol/μmol x 10^2^) (Fig 2D).

**Fig 3:**
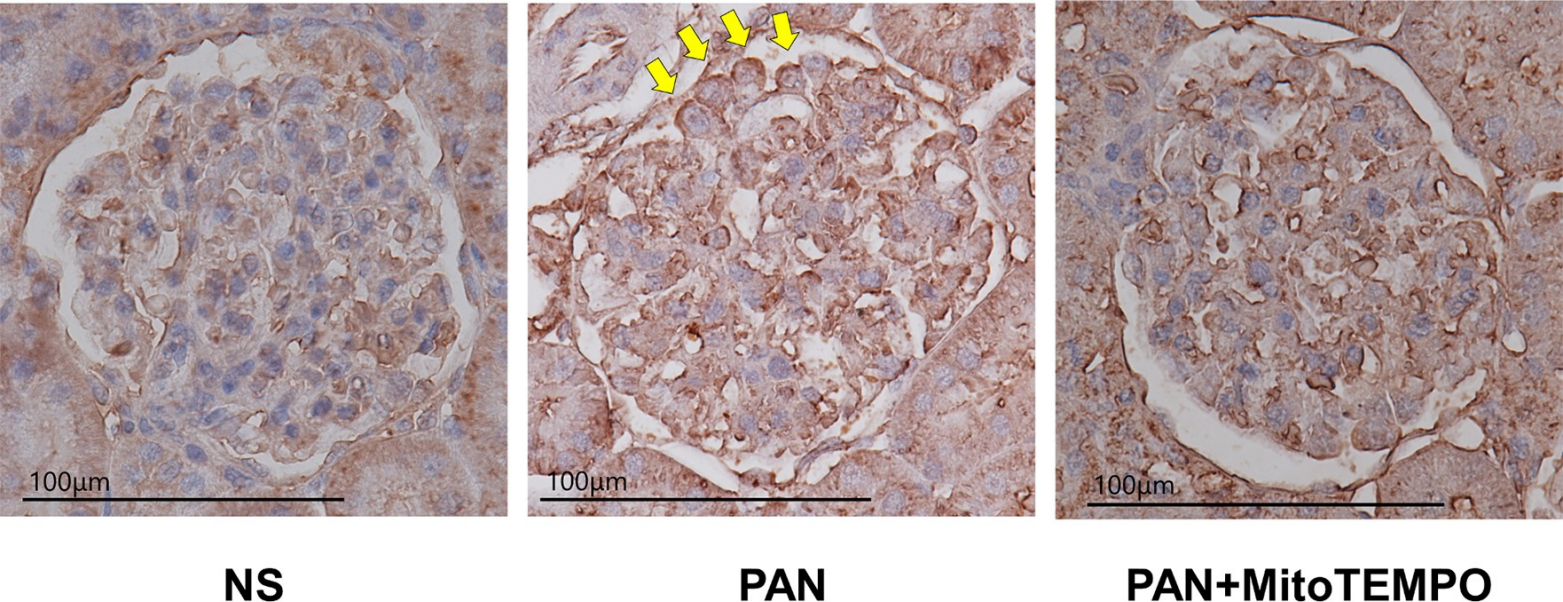
Representative images of 4HNE staining in the NS, PAN, PAN + MitoTEMPO groups. Anti-4HNE monoclonal antibody staining in podocytes was evident in the PAN group (as indicated by yellow arrows). PAN = puromycin aminonucleoside; 4HNE = 4-hydroxynonenal; NS = normal saline.

We detected anti-4HNE monoclonal antibody staining in podocytes in the PAN group. Representative images of samples in each group are shown in Fig 3.

### Light microscopy evaluation of the kidney tubulointerstitium, glomeruli, and podocyte apoptosis

**Fig 4:**
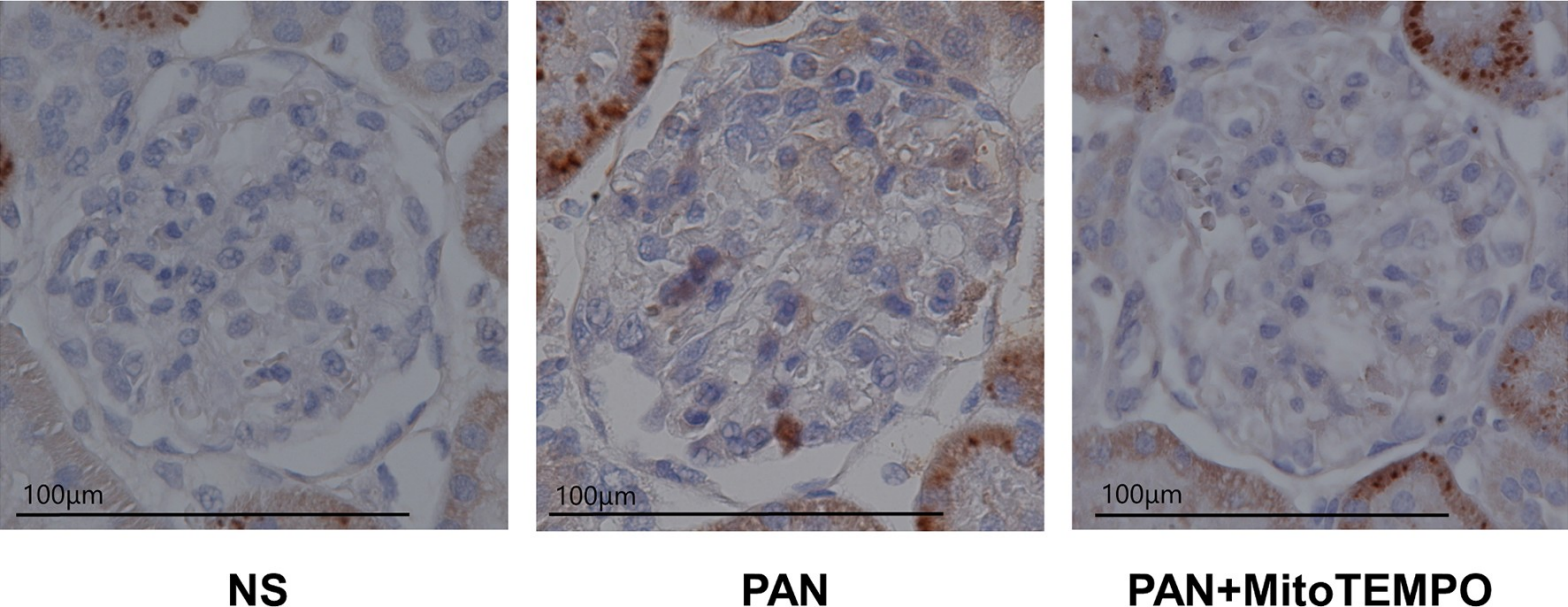
Representative images of staining for cleaved caspase-3 in the NS, PAN, PAN + MitoTEMPO groups. As far as we could observe in the slide samples using light microscopy, all glomeruli in each group were photographed to count the number of cells positive for cleaved caspase-3 in each glomerulus. The mean number of such cells was 0.051, 0.33 and 0.14 in the NS, PAN and PAN + MitoTEMPO groups, respectively. PAN = puromycin aminonucleoside; NS = normal saline.

Histological evaluation using light microscopy revealed only minor glomerular abnormalities and no major damage to the kidney tubulointerstitium. The mean number of cells positive for cleaved caspase-3 per glomerular section was 0.051, 0.33, 0.14 in the NS, PAN, and PAN + MitoTEMPO groups, respectively. Representative images of staining for cleaved caspase-3 in each group are shown in Fig 4.

### Evaluation by electron microscopy

**Fig 5:**
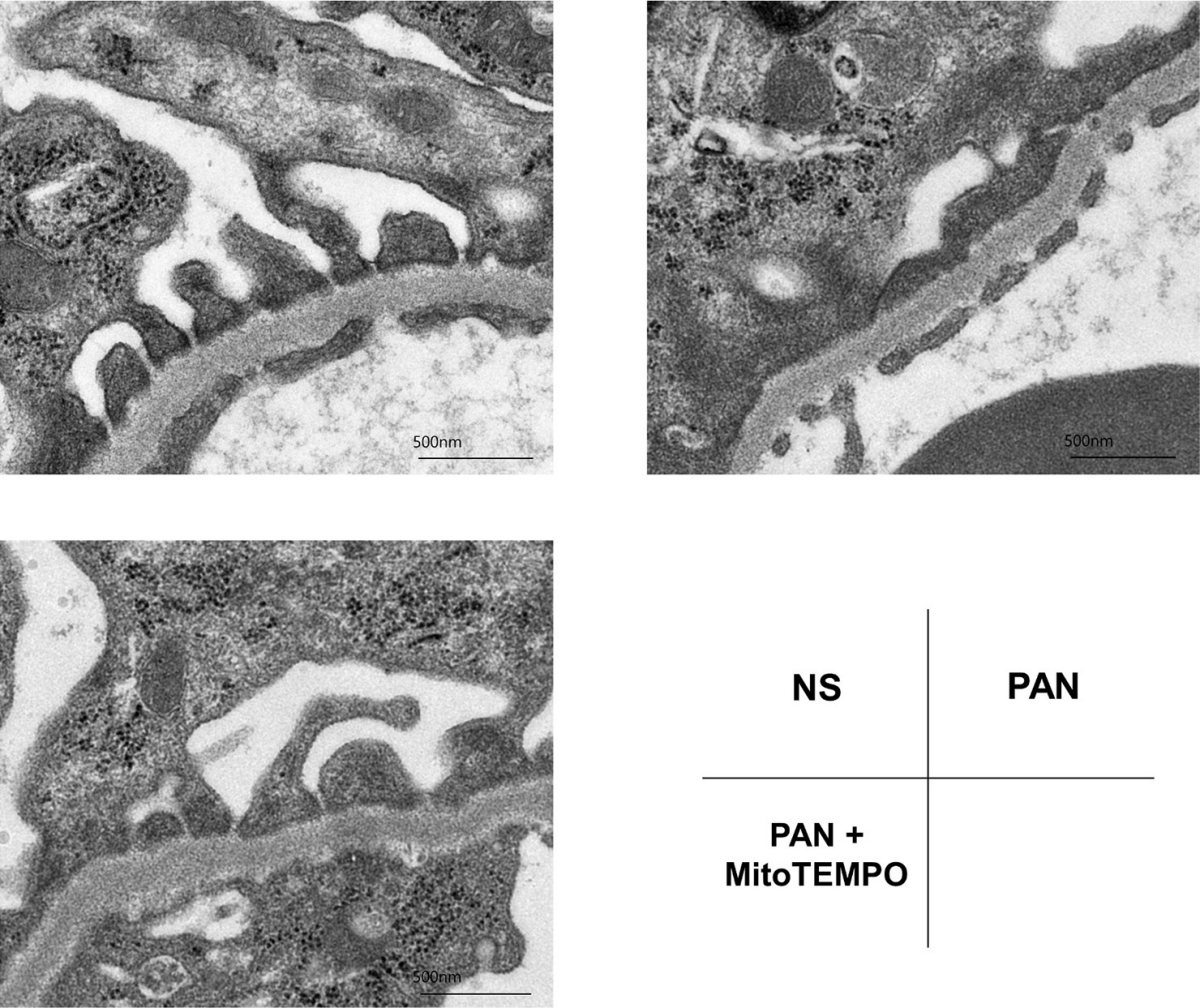
Representative images of podocyte foot processes in the NS, PAN, PAN + MitoTEMPO groups. Some of the left kidney samples from two randomly chosen different rats in the PAN group and PAN + MitoTEMPO group, and a single rat in the NS group were observed by transmission electron microscopy. Podocyte foot process effacement was evident in the PAN group and partially evident in the PAN + MitoTEMPO group. NS = normal saline; PAN = puromycin aminonucleoside.

Effacement of podocyte foot processes was evident in the glomerular sample from the PAN group, and also partially evident in the PAN + MitoTEMPO group (Fig 5). The daily urinary protein level and the foot process effacement in the PAN group were consistent with PAN nephrosis; a higher mean mitochondrial damage score in podocytes was correlated with more evident foot process effacement relative to other areas.

**Fig 6:**
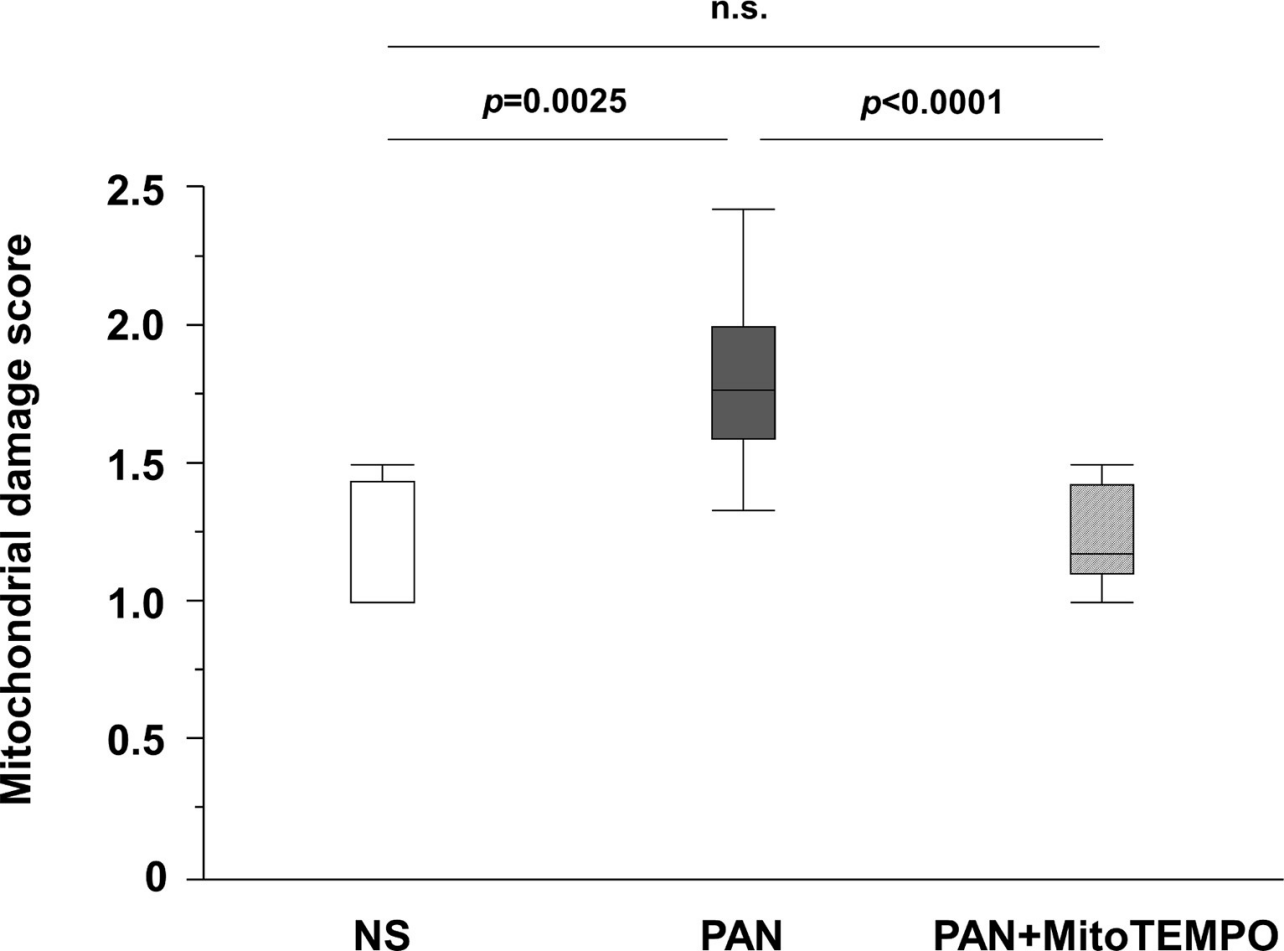
Mitochondrial damage score. We evaluated the mitochondrial damage score for all mitochondria observed in 5–18 randomly chosen podocytes in each group using electron microscopy. The mitochondrial damage score was rated as 1–5 based on ultrastructurally evident morphological damage resulting from the mitochondrial autophagy pathway. An intact mitochondrion was scored as 1 and mitochondrial autophagy was scored as 5. We scored the degree of mitochondrial damage for all visible mitochondria (n = 4–41 per podocyte) and calculated the average score for each podocyte. The average of the mitochondrial damage score in each podocyte was significantly lower in the PAN + MitoTEMPO group than in the PAN group. Data are expressed as the median and inter-quartile range. PAN = puromycin aminonucleoside; NS = normal saline; n.s. = not significant.

**Fig 7:**
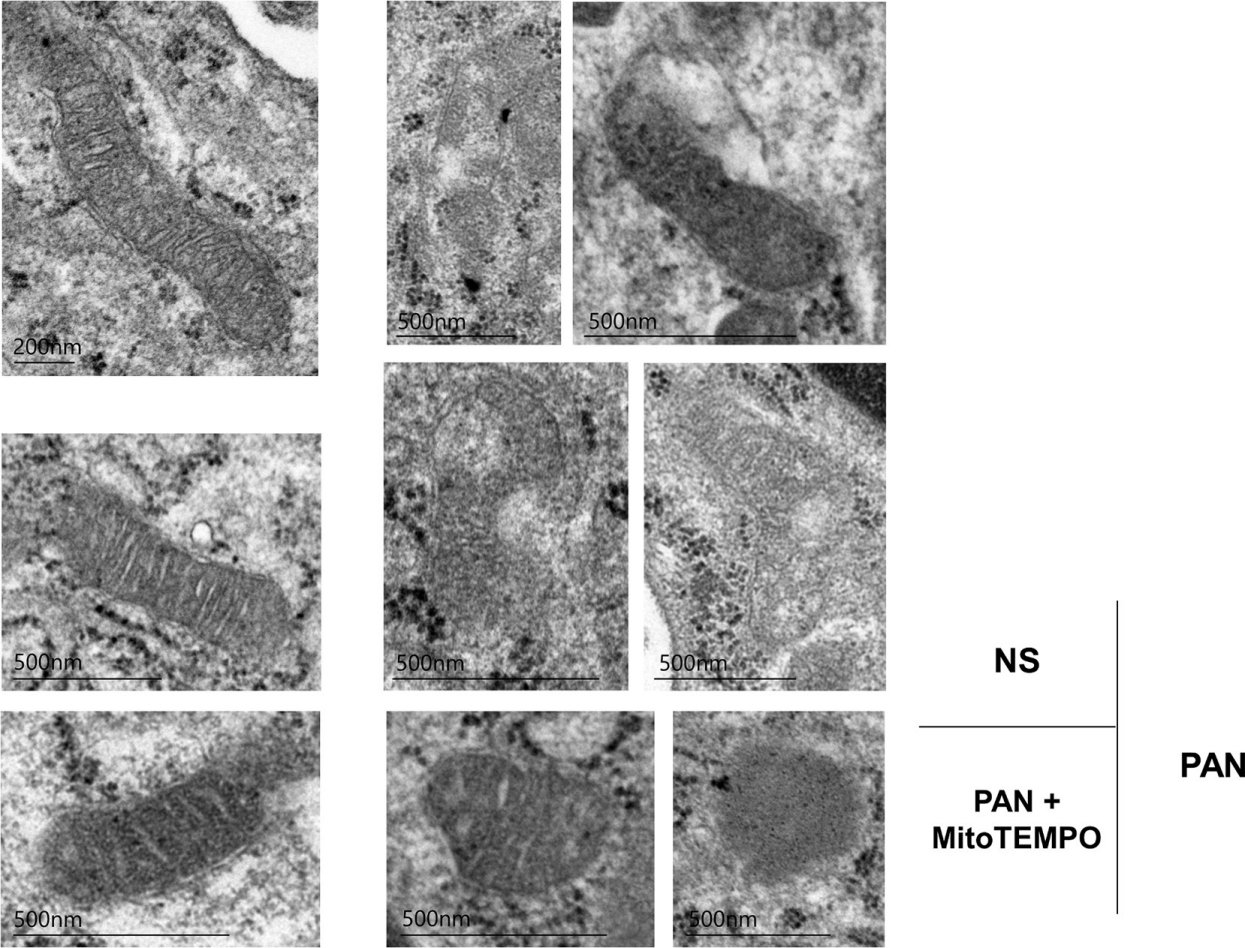
Representative images of mitochondria in the NS, PAN, and PAN + MitoTEMPO groups. Mitochondrial morphological changes in podocytes due to PAN and MitoTEMPO were observed by electron microscopy. In the PAN group, some of the mitochondrial cristae were absent, the matrix density was low in some areas, and the outer and inner membranes were separated. However, such features were not evident in the PAN + MitoTEMPO group. NS = normal saline; PAN = puromycin aminonucleoside.

The mitochondrial damage score was significantly lower (*p* <0.0001) in the PAN + MitoTEMPO group (1.2, IQR 1.1–1.4) than in the PAN group (1.8, IQR, 1.6–2.0) (Fig 6). Some representative images of mitochondria in each group are shown in Fig 7. Mitochondria assigned a worse score were more evident in the PAN group than in the other groups.

## Discussion

### Relationship between PAN-induced MCNS and mitochondrial ROS

Minimal-change disease is a major type of nephrotic syndrome in children, and a well-established rat model of MCNS induced by a single injection of PAN is available. As Gwinner *et al*. have reported that ROS production in glomeruli continues until 9 days after a single PAN injection [25], many researchers have investigated PAN-induced MCNS in relation to ROS production [25, 26]. This has led to a search for antioxidant agents that can ameliorate the disease state of PAN-induced MCNS, and several ROS scavengers have been reported [26]. We have also shown that α-tocopherol and edaravone are promising antioxidants that can ameliorate PAN-induced MCNS *in vivo* [27, 28]. More recently, it has become clear that other kidney diseases appear to be associated with mitochondrial damage and ROS.

While few reports have focused on minimal-change disease, Shen *et al*. showed that mitochondrial damage was ameliorated in murine podocytes cultured with PAN after pretreatment with cyclosporin A and tacrolimus [31]. Shen’s work and a few other studies have suggested that, *in vitro*, mitochondrial damage leads to podocyte apoptosis, suggesting that the latter could lead to proteinuria *in vivo* [31, 32]. However, whether or not mitochondrial damage actually occurs in an experimental animal model of MCNS, such as that induced by PAN, and the degree to which urinary protein can be decreased by mitochondrial scavenging therapy are issues that have remained unclear.

Here, using a single *in vivo* experimental model, we investigated the degree to which a mitochondrion-targeting ROS scavenger, MitoTEMPO, was able to ameliorate mitochondrial damage and reduce mitochondrial ROS and daily urinary protein excretion. As shown in Fig 1, the daily urinary protein excretion in the PAN + MitoTEMPO group was significantly decreased relative to the PAN group. The antioxidative effect of MitoTEMPO was equivalent to that of edaravone, which we had reported previously [28]. Fig 2 shows that the levels of TBARS in urine on day 9, TBARS in glomeruli, and 4HNE at sacrifice were significantly decreased in the PAN + MitoTEMPO group relative to the PAN group. Immunohistochemical staining demonstrated more frequent 4HNE staining of podocytes and podocyte apoptosis in the PAN group, consistent with previous reports and our assumptions. Thus, it was proved that specific scavenging of mitochondrial ROS by MitoTEMPO led to a decrease of both ROS production and daily urinary protein excretion.

Our next step was to demonstrate that not only was mitochondrial ROS scavenged by MitoTEMPO in the mitochondrial matrix, but also that mitochondrial damage was related to the increase of mitochondrial ROS and urinary protein. Therefore, using transmission electron microscopy, we studied the morphology of mitochondria to assess the degree of mitochondrial damage in podocytes.

### PAN-induced MCNS is related to mitochondrial damage

Only a few studies have attempted to assess mitochondrial morphology quantitatively in the kidney using electron microscopy [14, 35]. Sweetwyne *et al*. observed mitochondria in podocytes of aged mice with or without antioxidant therapy and classified the degree of mitochondrial damage using a score of 1–5 (a higher number indicating worse damage) [35]. Using this scoring method, we demonstrated that the mitochondrial damage in podocytes was significantly less severe in the PAN + MitoTEMPO group than in the PAN group, proving that MitoTEMPO reduced the amount of mitochondrial ROS, the extent of mitochondrial damage in podocytes, the degree of foot process effacement, and excretion of urinary protein.

We did not directly measure mitochondrial ROS or ROS only in podocytes. However, as the ROS surge in podocytes led to MCNS, and MitoTEMPO is a radical scavenger specific to mitochondria and reduces the degree of mitochondrial damage, it appears that podocyte mitochondrial ROS and subsequent damage are related to the disease state in MCNS. We suggest that the course of events in the PAN + MitoTEMPO group was as follows: (i) PAN caused a ROS surge in podocytes leading to foot process effacement, (ii) MitoTEMPO was taken up by podocyte mitochondria, where it reduced ROS, and (iii) the amelioration of mitochondrial damage contributed to foot process recovery and reduction of urinary protein excretion. The action of MitoTEMPO in preventing mitochondrial ROS production and damage may have been due to its accumulation in the mitochondrial matrix, where many mechanisms related to production of ROS operate [36–41], thus allowing MitoTEMPO to suppress them.

In conclusion, we have demonstrated that use of an antioxidant agent specific for mitochondria is able to reduce the amount of mitochondrial ROS, ameliorate mitochondrial damage in podocytes and reduce daily urinary protein excretion in an *in vivo* MCNS model. As the present study has revealed that mitochondrial oxidative stress plays a role in the disease state of MCNS, we conclude that this type of mitochondrion-targeting radical scavenger may have considerable utility for reducing both mitochondrial damage and urinary protein excretion in MCNS.
